# Stellate Ganglion Block for Post-acute Sequelae of SARS-CoV-2 Infection With Coexisting Post-traumatic Stress Disorder, Anxiety, and Chronic Pain: A Case Report

**DOI:** 10.7759/cureus.111897

**Published:** 2026-07-01

**Authors:** Maanas Chiplunkar, Joseph Kastrenakes, Ruben Dovlatyan, Sidharth Sahni, Michael Flamm

**Affiliations:** 1 Department of Physical Medicine and Rehabilitation, New York University, New York, USA; 2 Department of Anesthesiology, Division of Pain Medicine, New York University, New York, USA

**Keywords:** anxiety, dysautonomia, long covid, pasc, ptsd, stellate ganglion block

## Abstract

Post-acute sequelae of SARS-CoV-2 infection (PASC), commonly referred to as long COVID, is characterized by persistent symptoms, including fatigue, cognitive dysfunction, pain, and neuropsychiatric complaints, that can impair daily functioning. Proposed mechanisms are heterogeneous and may include autonomic dysregulation in a subset of patients. Stellate ganglion block (SGB) is a cervical sympathetic block used to treat symptoms of post-traumatic stress disorder (PTSD), anxiety, and, more recently, PASC. A 58-year-old man with a history of attention-deficit/hyperactivity disorder, generalized anxiety disorder, obsessive-compulsive disorder, autism spectrum disorder, PTSD, and chronic musculoskeletal pain developed persistent post-COVID symptoms following infection in March 2020, including brain fog, slowed information processing, word-finding difficulty, worsening anxiety, impaired balance, and reduced hand-eye coordination that significantly impaired occupational functioning as a chef. In January 2023, sequential left- and right-sided SGBs one week apart were performed to address cognitive dysfunction, fatigue, pain, and anxiety. Follow-up documentation described marked improvement in anxiety, frustration tolerance, and cognition lasting approximately six months. Repeat right-sided SGBs were performed in December 2024 and September 2025 for recurrence of symptoms and were again associated with symptomatic improvement. This is the first case demonstrating both longitudinal and reproducible functional improvement in cognitive symptoms, anxiety, and pain following serial SGBs in a patient with PASC complicated by psychiatric comorbidity and chronic pain. Although emerging observational literature suggests potential benefit of SGB in selected patients with PASC, prospective controlled studies are required to determine efficacy, mechanisms, and long-term outcomes.

## Introduction

Post-acute sequelae of SARS-CoV-2 infection (PASC), often referred to as long COVID, is characterized by persistent symptoms following recovery from acute COVID-19 infection. Patients frequently report fatigue, cognitive dysfunction (“brain fog”), pain, sleep disturbances, and neuropsychiatric symptoms that often impair daily functioning. Early outpatient data from the United States showed that a substantial proportion of patients had not returned to their usual state of health two to three weeks after testing positive for SARS-CoV-2, including individuals who were not hospitalized [1]. The pathophysiology of PASC remains incompletely understood and is likely multifactorial. Persistent inflammation, immune dysregulation, microvascular injury, and autonomic nervous system dysregulation are all implicated in its pathophysiology. Dysautonomia and sympathetic overactivity have been proposed as potential contributors to fatigue, cognitive impairment, and neuropsychiatric symptoms in some patients with PASC [2].

Stellate ganglion block (SGB) is a cervical sympathetic block traditionally used to treat sympathetically mediated pain syndromes affecting the head, neck, and upper extremity. The procedure may be performed with fluoroscopic or ultrasound guidance to visualize surrounding vascular and soft-tissue structures, and has a generally favorable safety and risk profile [3].

SGB has also been investigated as a neuromodulatory treatment for trauma-related and anxiety disorders. Because of the symptomatic and potential pathophysiologic overlap with conditions involving autonomic dysregulation and heightened sympathetic activity, SGB has been explored as a potential treatment for patients with PASC. Early reports in the literature include case series and retrospective cohort studies describing symptomatic improvement following treatment [4-6]. Consequently, although preliminary findings are encouraging, the efficacy, optimal patient selection, mechanisms of action, and durability of response remain incompletely defined. This report describes a patient with persistent PASC symptoms who experienced reproducible improvement following serial SGBs.

This work was previously presented as a meeting abstract at the New York New Jersey Pain Medicine Symposium on November 8, 2025.

## Case presentation

A 58-year-old man and a six-year US naval veteran presented with persistent symptoms consistent with PASC following COVID-19 infection in March 2020. Prior psychiatric history was notable for attention-deficit/hyperactivity disorder (ADHD) diagnosed at age 38, generalized anxiety disorder, obsessive-compulsive disorder, autism spectrum disorder, and post-traumatic stress disorder (PTSD). Additional medical history included chronic bilateral hip and shoulder pain for more than 10 years, hyperlipidemia, obstructive sleep apnea treated with continuous positive airway pressure, nephrolithiasis, vitamin D deficiency, and microscopic hematuria. Previous treatments for his chronic pain included greater trochanteric bursa and piriformis injections. His medications included amphetamine/dextroamphetamine for ADHD and hydroxyzine for anxiety. He had previously used escitalopram, which was discontinued after improvement following a change in occupation, and later restarted after anxiety symptoms resurfaced. He also participated in individual cognitive behavioral therapy for anxiety and an ADHD group therapy program.

During his acute COVID-19 illness in March 2020, he experienced anosmia, ageusia, and low-grade fever without respiratory compromise. SARS-CoV-2 antibody testing in October 2020 was positive. Following infection, he developed persistent cognitive and neuropsychiatric symptoms, including cognitive slowing (“brain fog”), difficulty processing information, word-finding difficulty, worsening anxiety related to workplace COVID-19 safety concerns, impaired balance, and reduced hand-eye coordination. These symptoms substantially impaired his ability to function as a chef, increasing the time required to complete routine tasks and contributing to significant distress. Over time, he was diagnosed with PTSD related to experiences working in a hospital environment during the pandemic.

Procedure technique

SGBs were performed under ultrasound guidance. The patient was positioned in the supine position to allow access to the cervical region. After sterile preparation and draping, a high-frequency linear ultrasound transducer was used to identify the C6 transverse process (Chassaignac tubercle), longus colli muscle, carotid artery, internal jugular vein, and surrounding soft-tissue structures. A 25-gauge needle was advanced under real-time ultrasound guidance toward the fascial plane adjacent to the longus colli muscle at the level of the stellate ganglion. After negative aspiration, 6 mL of 2% lidocaine was injected with visualization of appropriate spread along the prevertebral fascia. If indicated, the procedure was then repeated on the contralateral side, one week later, to complete the sequential bilateral blockade.

Intervention and outcomes

**Figure 1:**
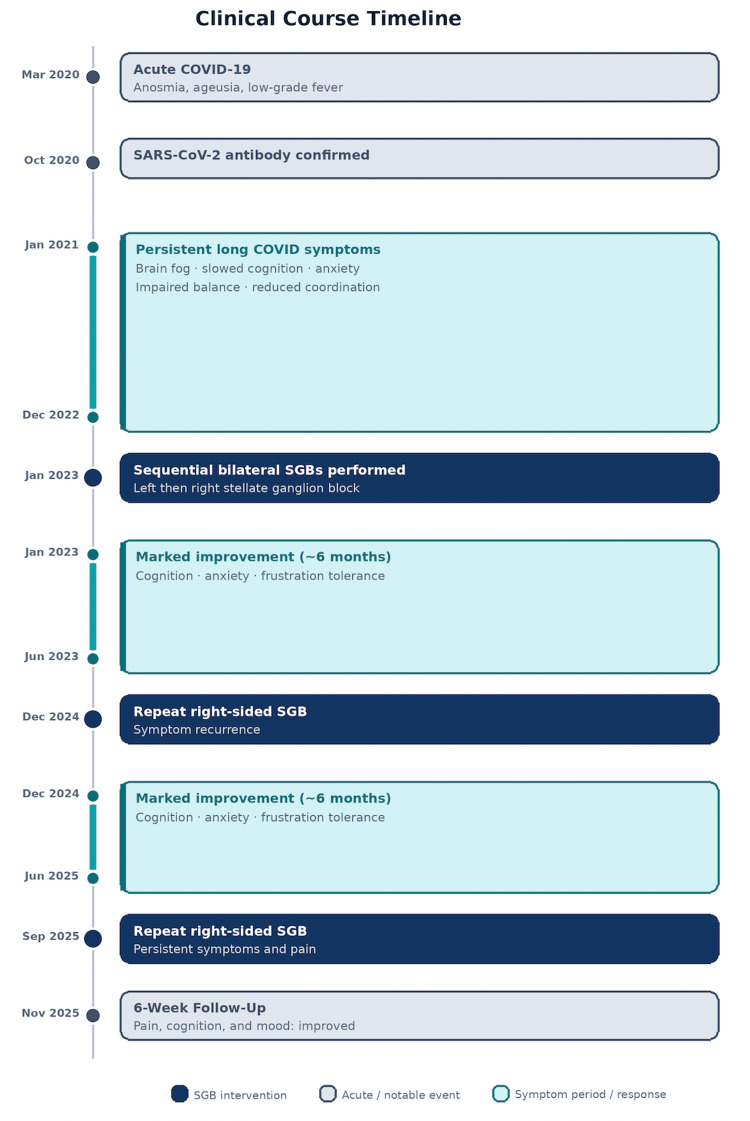
Clinical course timeline. SGB = stellate ganglion block

In January 2023 (34 months after the initial infection), sequential left- and right-sided SGBs one week apart were performed to address PASC symptoms. The patient tolerated the procedures without complication and returned to normal activities. He described marked improvement in anxiety, increased frustration tolerance, and improved cognition, with symptomatic relief lasting approximately six months (Figure 1).

In December 2024 (45 months after the initial infection, 23 months after the first SGB), a repeat right-sided SGB was performed for recurrent PASC symptoms affecting concentration and sensory complaints, including right-hand paresthesia involving the ring and small fingers and left thigh paresthesia. The patient described overall improvement, although symptom-specific magnitude and duration were not quantified (Figure 1).

In September 2025 (54 months after the initial infection, 20 months after the first SGB, 9 months after the second SGB), a right-sided SGB was performed with the primary goal of pain reduction in the context of persistent PASC symptoms. Head pain and bilateral hip pain improved immediately from 2/10 to 1/10. Across procedures, the patient consistently reported symptomatic improvement, although the degree and durability varied (Figure 1, Table 1).

**Table 1:** Outcomes after the September 2025 right-sided SGB. SGB = stellate ganglion block; NRS = Numeric Rating Scale [7]

Outcome	Pre-SGB	Immediate post-procedure	Follow up (6 weeks)
Head pain (NRS)	2/10	1/10	Improved
Hip pain (NRS)	2/10	1/10	Improved
Cognitive function	Brain fog, slowed processing	Improved clarity	Sustained improvement ~6 months after the initial SGB
Anxiety/Frustration tolerance	Severe anxiety, low frustration tolerance	Improved	Continued improvement noted

## Discussion

This case describes reproducible and longitudinal symptomatic improvement following SGB in a patient with PASC complicated by PTSD, anxiety, and chronic musculoskeletal pain. Notably, the patient experienced clinically meaningful improvement in cognitive function, emotional regulation, and pain following sequential bilateral SGB, with the most durable benefit occurring after the initial treatment and a reproducible response after subsequent unilateral blocks for symptom recurrence.

These findings are consistent with emerging, though still preliminary, literature evaluating SGB in PASC populations. Liu and Duricka first reported improvement in persistent PASC symptoms following sequential bilateral SGB in a small case series, proposing dysautonomia as a potential contributor to symptom persistence [4]. Subsequent observational studies have suggested that these findings may extend beyond isolated cases. Pearson et al. reported a retrospective cohort of 41 patients with PASC, in which 86% experienced improvement in at least one symptom following SGB [5]. Additional retrospective and observational studies have described improvements in fatigue, cognitive dysfunction, sleep disturbance, and mood-related symptoms following cervical sympathetic blockade [8-10]. While these studies are limited by design and heterogeneity, they collectively support the hypothesis that modulation of the sympathetic nervous system may influence symptom burden in selected PASC phenotypes.

Evidence from PTSD literature provides additional context for the potential neuromodulatory effects of SGB, although results remain mixed. Mulvaney et al. reported clinically meaningful improvement in PTSD symptoms in more than 70% of patients in a large case series of combat-related PTSD [6]. However, randomized controlled trials have produced variable findings. Hanling et al. demonstrated improvement in both treatment and sham groups without a statistically significant difference, suggesting a possible placebo or contextual effect [11]. In contrast, a multicenter randomized clinical trial by Rae Olmsted et al. found that paired SGB treatments resulted in significantly greater reductions in PTSD symptom severity compared with sham treatment over an eight-week period [12]. A recent systematic review and meta-analysis concluded that while SGB may reduce PTSD symptom severity, the limited number of high-quality randomized trials constrains definitive conclusions [13].

SGB has also demonstrated potential benefits in anxiety populations. In a large case series of 285 patients, Lynch et al. reported substantial reductions in generalized anxiety symptoms, with mean Generalized Anxiety Disorder-7 scores decreasing significantly at one month following treatment [14].

The mechanism by which SGB may influence PASC symptoms remains incompletely understood. PASC is increasingly recognized as a heterogeneous condition involving overlapping mechanisms, including immune dysregulation, persistent inflammation, microvascular dysfunction, and autonomic nervous system imbalance [2,15]. Autonomic dysfunction, particularly sympathetic overactivity, has been proposed as a contributor to fatigue, cognitive dysfunction, and neuropsychiatric symptoms in a subset of patients [2]. SGB produces a temporary interruption of cervical sympathetic outflow and may modulate central autonomic networks involved in stress response, arousal, and inflammatory signaling [16]. One hypothesis is that SGB may disrupt a maladaptive sympathetic feedback loop contributing to persistent autonomic dysregulation in PASC, thereby facilitating the restoration of autonomic balance and improvement in cognition.

Although symptomatic improvement was observed, the ability to attribute causality to SGB remains limited by potential placebo and contextual effects, as well as the fluctuating and often relapsing-remitting natural course of PASC. In addition, this case lacks blinding and placebo control, introducing possible expectation and observer biases. The absence of standardized, objective outcome measures, such as PROMIS domains, GAD-7, or validated cognitive assessments, limits the ability to quantify the magnitude and reproducibility of response across interventions. As a single-patient case report, these findings are inherently limited in generalizability and may not reflect responses in broader or more heterogeneous PASC populations.

To the author’s knowledge, published literature has only described short-to-intermediate-term symptomatic improvement after single-course or short-interval repeat SGB in PASC, while detailed reports of recurrent benefit across serial SGBs over a multi-year course remain limited. Early published evidence of longitudinal benefit includes the 2022 case series by Liu and Duricka, which described two patients with durable symptomatic improvement through 60-day follow-up after sequential bilateral SGB [4]. Duricka and Liu’s 2024 retrospective review reported durable responders at one month, with some patients later pursuing additional SGB treatment after recurrent symptoms, while Levey et al. reported a maintained symptomatic relief for 6-11 weeks in a subset of patients after treatment [8,17]. More recent reviews likewise characterize the existing evidence base as promising but limited by small, heterogeneous, and largely uncontrolled studies, with benefit generally described over weeks to months rather than across an extended serial-treatment course [18]. Based on the available literature, this case report is the first of its kind to describe serial repeat SGB interventions administered over a span of nearly two years for recurrent PASC symptoms, with recurrent symptomatic benefit after each treatment.

## Conclusions

This case demonstrates reproducible and sustained symptomatic improvement following serial SGB in a patient with PASC, including cognitive, emotional, and pain-related symptoms. Notably, it represents the first report to describe longitudinal SGB administration over a multi-year period with consistent therapeutic response to repeat interventions for recurrent symptoms. While prior literature has described short-to-intermediate-term benefits after limited treatment courses, this case highlights the potential for durable and recurrent benefit across serial treatments. While these observations support the hypothesis that sympathetic modulation may play a role in select PASC phenotypes, they should be considered exploratory and interpreted within the limitations inherent to a single-patient case report. Although emerging literature suggests potential benefit of SGB in selected patients with PASC, prospective controlled studies are required to determine efficacy, mechanisms, optimal patient selection, and long-term outcomes.
